# Effectiveness of current policing-related mental health interventions in England and Wales and Crisis Intervention Teams as a future potential model: a systematic review

**DOI:** 10.1186/s13643-017-0478-7

**Published:** 2017-04-17

**Authors:** Eddie Kane, Emily Evans, Farhad Shokraneh

**Affiliations:** 1 Institute of Mental Health, Centre for Health and Justice, University of Nottingham, Nottingham, UK; 2 School of Medicine, University of Nottingham, Nottingham, UK; 3 Institute of Mental Health, University of Nottingham, Nottingham, UK

**Keywords:** Police, Mental health, Custody, Triage, Diversion, Crisis intervention training

## Abstract

**Background:**

Experiencing mental ill health adds a layer of complexity for individuals in touch with the justice system and for those responsible for working in the justice service with these individuals, such as frontline police officers.

In England and Wales, there are three commonly used but not necessarily commonly designed or operated, mental health interventions associated with policing, Liaison and Diversion, Street Triage and specialist staff embedded in Police Contact Control Rooms. A fourth US designed model, Crisis Intervention Teams (CITs), is now attracting some interest in England and Wales, and these four are to be considered in this review. A fifth intervention, Mental Health Courts, was trialed but has now been abandoned in England and Wales and so has been excluded, but remains in use elsewhere.

In recent years, there has been an increase in the level of investment related to these intervention options. This has largely been without an evidence base being available to aid design, structure, and consistency of approach. The review will address this gap and provide a systematic review of each of these options. This will provide a baseline of research evidence for those who commission and provide services for individuals experiencing mental ill health and who are in contact with the justice system.

**Methods:**

Twenty-nine relevant databases and sources have been selected which will be systematically searched to locate relevant studies. These studies have to meet the set inclusion criteria which require them to report an objective outcome measure(s) in respect of offending or mental health outcomes and to have an experimental or quasi-experimental design including a comparator group(s) or a pre/post comparison. The review will exclude PhD theses, papers in non-English languages and papers published prior to 1980.

Keywords have been collected through canvassing experts’ opinion, literature review, controlled vocabulary and reviewing the results of a primary scoping review carried out to aid the development of the PICO, composed of Population/Participants, Intervention/Indicator, Comparator/Control, and Outcomes. For the proposed review, the key elements of the PICO are the following: persons with mental health problems, symptoms or diagnoses who come into contact with the police; interventions involving partnership working between police and mental health nurses and related professionals to divert those with mental health problems away from criminal justice processes; comparisons with control groups or areas where such interventions have not been introduced; and outcomes concerning criminal justice and health outcomes.

The results of the searches will be screened using the set criteria and the selected papers reviewed and analysed to allow findings regarding these interventions to be reported.

**Discussion:**

The objectives of the review are firstly to identify and report research on the relevant interventions, nationally and internationally and then secondly to consider, when possible, which interventions or aspects of those interventions are effective. This is judged with regard to changes in mental health status or service use and future offending behaviour.

The approaches to be considered have gained a good deal of support and funding over recent years, and this review will provide a systematic review of the underpinning research evidence to inform future commissioning, service design and investment decisions.

**Electronic supplementary material:**

The online version of this article (doi:10.1186/s13643-017-0478-7) contains supplementary material, which is available to authorized users.

## Background

One in four people experience mental health problems in any given year, and many will come into contact with the police either as victims of crime, witnesses, offenders or when detained under Section 136 of the Mental Health Act [1].

Mental ill health and related conditions add a layer of complexity and challenge for individuals in touch with the justice system and for those responsible for delivering justice services to them, including police. Individuals with mental health problems are more likely to be victims of crime than others [2]. It is estimated that worldwide there is an overall prevalence of 3.7% of male and female prisoners with a psychotic illness, and 11.4% with major depression, levels which have not materially changed since a 2002 review [3]. The Adult Psychiatry Morbidity Survey of households in England reports that of prisoners surveyed, anti-social personality disorder (ASPD) was reported in a very high proportion of inmates: 63% of male remand prisoners and 49% of male sentenced prisoners, with people with the disorder accounting for a disproportionately large proportion of crime and violence committed [1]. Similarly, 31% of younger people (aged 13–18) who offended (including young people in custody and in the community) were identified as having a mental health need [4].Learning disability is more common in young people in custody, a prevalence of 23–32%, compared to 2–4% of the general population [4, 5]. Furthermore, 31% of young offenders were assessed as ‘borderline’ regarding intellectual functioning as measured via the Wechsler Abbreviated Scale of Intelligence (WASI) [4].

Overall, individuals experiencing mental health problems often fair poorly in the justice system, including with the police [2]. The police frequently fulfil the role of gatekeeper in deciding whether a person with mental illness who has come to their attention should enter the mental health system or the criminal justice system. Criminalisation may result if this role is not performed appropriately [6]. Victims with SMI did report their experience to the police but were much less satisfied with them and less likely to report fair or respectful treatment [2]. Police interventions involving individuals with mental illness and who were suspected of minor offences were more likely to lead to their arrest. For offences of equal severity if the citizen involved had a mental illness, they were twice as likely as those involving individuals with no suggestion of mental illness, to lead to arrest [7].

Police officers’ encounters with people with mental illnesses can be particularly challenging to both parties. For the police, these encounters often take much more time than other calls for service, require officers to have special training and skills, typically involve repeat contacts with the same individuals, are mostly in response to a person with mental illness committing a minor or “nuisance” offence and occasionally involve volatile situations, risking the safety of all involved and their successful resolution may depend on the availability of community mental health resources [8].

In England, there are three commonly used but not necessarily commonly designed or operated interventions; Liaison and Diversion (L&D), Street Triage and specialist staff embedded in Police Contact Control Rooms (CCRs). Another and more integrated approach, Crisis Intervention Teams (CITs), based on the Memphis model from the USA will be included in this review as it is relatively better researched and there is some early interest amongst police forces. We do not suggest that it is an immediately transferrable model but its integrative approach may be of interest to commissioners, providers and users of current services. Mental health courts will be excluded from this review as they no longer operate in England and in addition tend to involve at most a very limited role for the police. More detail on the included interventions is provided below.

### Liaison and Diversion

These services aim to divert individuals at their earliest possible point of contact with the justice system. Teams of specialist mental health-trained staff are located at police custody suites or courts in order to assess and refer on to more appropriate mental health services outside the justice system. Alternatively, they may support the individual whilst they remain in the justice system if their index offence or risk means they cannot be diverted immediately.

### Street Triage

Street Triage involves a joint mental health service and policing approach to an individual in crisis or at risk. Based on locally agreed protocols, Street Triage aims to support access to appropriate crisis care and to provide more timely access to other health, social care, and third sector services.

### Embedded staff in CCRs

For a person in a mental health crisis, at times, the police contact control room can be their first point of contact. For the most part, the embedded staff are mental health professionals, although in a few forces they are augmented by paramedic professionals. These services are designed to help triage calls to CCRs that may be from individuals experiencing mental health problems to ensure that they get an appropriate response to their call.

### Crisis Intervention Teams

Not to be confused with Crisis Intervention Teams operated by local authority Social Services in England, the CIT model initially developed in 1988, following the fatal shooting of a man with a history of mental illness and substance abuse by a Memphis police officer. It involves specially trained police officers who respond to calls involving suspected mental ill-health either alone or alongside mental health and addiction professionals. The model intends to increase safety in encounters and when appropriate divert persons with mental illnesses from the criminal justice system to mental health treatment. These interventions combine elements of the commonly used UK interventions noted above, and a number of Police Forces are looking at the option of adopting this approach.

### Summary

In recent years, there has been an increase in the level of investment related to these intervention options but often with no apparent examination and application of the evidence base. This review will address this gap and provide a systematic review of each of the key options. This will provide a baseline of research evidence for those who commission and provide services for individuals experiencing mental ill health and who are in contact with the justice system.

## Methods

This review is concerned with interventions to improve the interactions between people with mental health problems and the police. This was operationalised using a Population/Participants, Intervention/Indicator, Comparator/Control, Outcomes (PICO) search framework, and the content of this framework for this review is outlined below:

### Population/participants

- Persons with mental health problems, symptoms and diagnoses who come into contact with the police (target population)
- Police officers and practitioners from other agencies who deal with the target population whether in person or via telephone contact
- Partner agencies: the range of mental health practitioners and agencies providing these services (adult mental health, personality disorder services, community forensic service, child and family services, drug and alcohol misuse services, community mental health teams, forensic outpatient units, etc.), probation, Youth Offending Teams (YOTs), court staff, magistrates and judges, lawyers, Crown Prosecution Service (CPS), social services, and health services

### Intervention/indicator

- Basing mental health practitioners in contact control rooms, police stations, custody suites, or embedding them with other police teams to ensure appropriate treatment and/or referral—including assessments
- Partnership working to divert those with mental health problems away from criminal justice processes (e.g. when suspected of committing an offence, voluntary attendance, arrest, detention in custody), where appropriate or to ensure appropriate treatment, of whatever sort (e.g. hospitalisation) or support within the justice system
- Or any similar approaches with similar aims

### Comparator/control

- These interventions have been introduced in different phases across the country, providing opportunities for comparison, although different combinations may be in place in different areas.
- The alternative would be other designs such as time-series (before-and-after) comparisons if this is not the case in other locations.

### Outcome(s)

These have been divided between primary, which concern outcomes likely to be directly affected by the interventions, and secondary on which the interventions may have an effect but which may also be affected by other factors.

Primary:

- Improved assessment, referral and treatment (quality and timeliness) of those with mental illness
- Reduced demand on police forces and police officer time
- Improved mental health outcomes (such as change in status or diagnosis as assessed by practitioners using valid measures) and level of service engagement
- Reduced use of Section 135/6 of the Mental Health Act

Secondary:

- Increased demand on community mental health services
- Reductions in reoffending or arrest

These will be assessed using the outcome measurements in the selected papers.

The elements of this PICO framework are broken out into search terms to conduct the review (these are listed in Appendix 1 for each database/source searched).

The criteria used to select studies for inclusion will be as follows:

- Does the study have an objective outcome measure(s) regarding offending or health?
- Does the study focus on males and or females aged over 18?
- Does the study focus on a mental health intervention?
- Does the paper focus on people with a mental illness/mental health problem?
- Is the paper experimental or quasi-experimental?
- Does the study include an intervention and comparator group(s), or does it contain a pre/post design?
- Is the comparator group individually matched to intervention participants or is baseline comparability demonstrated or is there random allocation of participants?

The review will exclude sources which are PhD theses (for reasons of research rigor and quality), published in non-English language (for reasons of time and resources) or published prior to 1980 (the services in the format that this review deals with were not available prior to 1980). These criteria will be used to select papers for inclusion in the review. Studies of varying quality will be retaining in the review and differentiated in the discussion.

Twenty-nine databases and sources will be searched electronically using the strategies laid out in Appendix 1. Search results will be presented and managed in EndNote X7. This will be used to undertake the initial screen of the results, based on title and abstract. A full text review of the remaining papers will then be undertaken. These processes will be undertaken by two researchers (Kane and Evans). Any disagreement will be resolved through discussion and resource to a third party. The review will report on the research aims, methodology and key findings of the papers included in the review. These will be presented in summary tables for each intervention type as well as overall regarding policing interventions for those with mental health issues.

It is expected that due to the limited and varied nature of the research evidence in this field, it will not be possible to quantitatively synthesise findings across the included studies in which case the findings will be narratively synthesised. Where the study data allows it, we will conduct data synthesis. For example, studies reporting the same outcome measures, such as number of arrests, days in jail (places of initial detention used in US studies) and days in treatment, will be compared and reported alongside each other. Studies reporting outcome measures on similar topics, such as recidivism or mental health treatment, will also be compared as far as possible.

In order to avoid publication bias and selective reporting, the search conducted will include grey literature and unpublished reports. Where the nature of the studies permit it, a GRADE table of findings will be developed in order to support the identification of relevant results with regard to the aims of the study, research design, and outcome measures. As above, the nature of the studies in the topic area may require such a table to be adapted. Relevant studies will be assessed for risk of bias using the Cochrane tools for RCT and non-RCT designs, RoB 2.0 and ROBINS-I respectively.^1^ These consider aspects of the study design and conduct. The authors will also assess the same outcomes from different studies together and contact the authors for more information if required as research protocols in this area do not always get formally registered.

This protocol has been registered in PROSPERO international prospective register of systematic reviews, registration number CRD42017057039.^2^ Details of this methodology are laid out in the associated PRISMA-P (please see Additional file 1 PRISMA-P).

## Discussion

This review will outline the evidence for four mental health interventions associated with policing, Liaison and Diversion, Street Triage, specialist staff embedded in Police Contact Control Rooms and Crisis Intervention Teams. This review will address the gap which currently exists regarding the operation and effectiveness of these interventions.

It will do this through a systematic search of relevant databases and resources using a PICO and set of inclusion/exclusion criteria to draw off relevant studies. The analysis of these will provide a baseline of research evidence for those who commission and provide services for individuals experiencing mental ill health and who are in contact with the justice system.

## Appendix 1

### Search Strategies

A. *Campbell Library*
  Coordinating groups: Crime and Justice
  Type of document: Title, Protocol, Review, User abstract, Other
B. *Cochrane Library (Including CDSR, CENTRAL, DARE, HTA, CMR, NHS EED)*
  1. (Accompan* or Collaborat* or Cooperat* or Engag* or Initiative* or Integrat* or Interact* or Liaison or Model* or Partners or Partnership* or Team*):ti,ab
  2. MeSH descriptor: [Crime] this term only
  3. MeSH descriptor: [Criminals] this term only
  4. MeSH descriptor: [Crisis Intervention] this term only
  5. MeSH descriptor: [Emergencies] this term only
  6. (Crime or Crisis or Crises or Event or Events or Occur* or Incident* or Emergenc* or Disturbance*):ti,ab
  7. #2 or #3 or #4 or #5 or #6
  8. MeSH descriptor: [Law Enforcement] this term only
  9. MeSH descriptor: [Police] this term only
  10. (Arrest* or Custody or "Law Enforcement" or Police or "Re-Arrest"):ti,ab
  11. #8 or #9 or #10
  12. MeSH descriptor: [Mental Disorders] explode all trees
  13. MeSH descriptor: [Mentally Ill Persons] explode all trees
  14. (“Mental Health Conditions” or “Mental Health Crisis” or “Mental Health Issues” or “Mental Illness” or “Mental Illnesses” or “Psychiatric Crisis” or “Psychiatric Emergencies” or “Psychiatric Emergency” or “Psychiatric Syndromes”):ti,ab
  15. #12 or #13 or #14
  16. #1 and #7 and #11 and #15
C. *EMBASE*
  1. (Accompan* or Collaborat* or Cooperat* or Engag* or Initiative* or Integrat* or Interact* or Liaison or Model* or Partners or Partnership* or Team*).ti,ab.
  2. Crime/OR Offender/OR Crisis Intervention/OR Emergency/OR (Crime or Crisis or Crises or Event or Events or Occur* or Incident* or Emergenc* or Disturbance*).ti,ab.
  3. Law Enforcement/OR Police/OR (Arrest* or Custody or “Law Enforcement” or Police or “Re-Arrest”).ti,ab.
  4. Exp Mental Disease/OR (“Mental Health Conditions” or “Mental Health Crisis” or “Mental Health Issues” or “Mental Illness” or “Mental Illnesses” or “Psychiatric Crisis” or “Psychiatric Emergencies” or “Psychiatric Emergency” or “Psychiatric Syndromes”).ti,ab.
  5. 1 AND 2 AND 3 AND 4
  6. Limit 5 to exclude MEDLINE journals
D. *MEDLINE via Ovid SP*
  1. (Accompan* or Collaborat* or Cooperat* or Engag* or Initiative* or Integrat* or Interact* or Liaison or Model* or Partners or Partnership* or Team*).ti,ab.
  2. Crime/OR Criminals/OR Crisis Intervention/OR Emergencies/OR (Crime or Crisis or Crises or Event or Events or Occur* or Incident* or Emergenc* or Disturbance*).ti,ab.
  3. Law Enforcement/or Police/or (Arrest* or Custody or “Law Enforcement” or Police or “Re-Arrest”).ti,ab.
  4. Exp Mental Disorders/OR Exp Mentally Ill Persons/OR (“Mental Health Conditions” or “Mental Health Crisis” or “Mental Health Issues” or “Mental Illness” or “Mental Illnesses” or “Psychiatric Crisis” or “Psychiatric Emergencies” or “Psychiatric Emergency” or “Psychiatric Syndromes”).ti,ab.
  5. 1 AND 2 AND 3 AND 4
E. *National Police Library Catalogue*
  All Fields: (Mental)
F. *ProQuest Databases*
  Databases - 8 databases searched:
  - Applied Social Sciences Index & Abstracts (ASSIA)
  - International Bibliography of the Social Sciences (IBSS)
  - National Criminal Justice Reference Service (NCJRS) Abstracts Database
  - PAIS Index
  - ProQuest Dissertations & Theses: UK & Ireland
  - ProQuest Dissertations & Theses A&I
  - Social Services Abstracts
  - Sociological Abstracts
  (ti(Accompan* OR Collaborat* OR Cooperat* OR Engag* OR Initiative* OR Integrat* OR Interact* OR Liaison OR Model* OR Partners OR Partnership* OR Team*) OR ab(Accompan* OR Collaborat* OR Cooperat* OR Engag* OR Initiative* OR Integrat* OR Interact* OR Liaison OR Model* OR Partners OR Partnership* OR Team*)) AND (ti(Crime OR Crisis OR Crises OR Event OR Events OR Occur* OR Incident* OR Emergenc* OR Disturbance*) OR ab(Crime OR Crisis OR Crises OR Event OR Events OR Occur* OR Incident* OR Emergenc* OR Disturbance*)) AND (ti(Arrest* OR Custody OR “Law Enforcement” OR Police OR “Re-Arrest”) OR ab(Arrest* OR Custody OR “Law Enforcement” OR Police OR “Re-Arrest”)) AND (ti(“Mental Health Conditions” OR “Mental Health Crisis” OR “Mental Health Issues” OR “Mental Illness” OR “Mental Illnesses” OR “Psychiatric Crisis” OR “Psychiatric Emergencies” OR “Psychiatric Emergency” OR “Psychiatric Syndromes”) OR ab(“Mental Health Conditions” OR “Mental Health Crisis” OR “Mental Health Issues” OR “Mental Illness” OR “Mental Illnesses” OR “Psychiatric Crisis” OR “Psychiatric Emergencies” OR “Psychiatric Emergency” OR “Psychiatric Syndromes”))
G. *PsycINFO via Ovid SP*
  1. (Accompan* or Collaborat* or Cooperat* or Engag* or Initiative* or Integrat* or Interact* or Liaison or Model* or Partners or Partnership* or Team*).ti,ab.
  2. Crime/OR Criminals/OR Crises/OR Emergency Management/OR Crisis Intervention/OR (Crime or Crisis or Crises or Event or Events or Occur* or Incident* or Emergenc* or Disturbance*).ti,ab.
  3. Law Enforcement/OR Police Personnel/OR (Arrest* or Custody or “Law Enforcement” or Police or “Re-Arrest”).ti,ab.
  4. Exp Mental Disorders/OR Mentally Ill Offenders/OR Psychiatric Patients/OR (“Mental Health Conditions” or “Mental Health Crisis” or “Mental Health Issues” or “Mental Illness” or “Mental Illnesses” or “Psychiatric Crisis” or “Psychiatric Emergencies” or “Psychiatric Emergency” or “Psychiatric Syndromes”).ti,ab.
  5. 1 AND 2 AND 3 AND 4
H. *PubMed Excluding MEDLINE*
  (Accompan*[tiab] OR Collaborat*[tiab] OR Cooperat*[tiab] OR Engag*[tiab] OR Initiative*[tiab] OR Integrat*[tiab] OR Interact*[tiab] OR Liaison[tiab] OR Model*[tiab] OR Partners[tiab] OR Partnership*[tiab] OR Team*[tiab]) AND (Crime[MeSH:NoExp] OR Criminals[MeSH:NoExp] OR Crisis Intervention[MeSH:NoExp] OR Emergencies[MeSH:NoExp] OR Crime[tiab] OR Crisis[tiab] OR Crises[tiab] OR Event[tiab] OR Events[tiab] OR Occur*[tiab] OR Incident*[tiab] OR Emergenc*[tiab] OR Disturbance*[tiab]) AND (“Law Enforcement”[MeSH:NoExp] OR Police[MeSH:NoExp] OR Arrest*[tiab] OR Custody[tiab] OR “Law Enforcement”[tiab] OR Police[tiab] OR “Re-Arrest”[tiab]) AND (“Mental Disorders”[MeSH] OR “Mentally Ill Persons”[MeSH] OR “Mental Health Conditions”[tiab] OR “Mental Health Crisis”[tiab] OR “Mental Health Issues”[tiab] OR “Mental Illness”[tiab] OR “Mental Illnesses”[tiab] OR “Psychiatric Crisis”[tiab] OR “Psychiatric Emergencies”[tiab] OR “Psychiatric Emergency”[tiab] OR “Psychiatric Syndromes”[tiab]) NOT MEDLINE[sb]
I. *Scopus*
  (TITLE-ABS-KEY (accompan* OR collaborat* OR cooperat* OR engag* OR initiative* OR integrat* OR interact* OR liaison OR model* OR partners OR partnership* OR team*) AND TITLE-ABS-KEY (crime OR crisis OR crises OR event OR events OR occur* OR incident* OR emergenc* OR disturbance*) AND TITLE-ABS-KEY (arrest* OR custody OR “Law Enforcement” OR police OR “Re-Arrest”) AND TITLE-ABS-KEY (“Mental Health Conditions” OR “Mental Health Crisis” OR “Mental Health Issues” OR “Mental Illness” OR “Mental Illnesses” OR “Psychiatric Crisis” OR “Psychiatric Emergencies” OR “Psychiatric Emergency” OR “Psychiatric Syndromes”))
J. *Web of Science*
  Databases:
  - Science Citation Index Expanded (SCI-EXPANDED) --1900-present
  - Social Sciences Citation Index (SSCI) --1956-present
  - Arts & Humanities Citation Index (A&HCI) --1975-present
  - Conference Proceedings Citation Index- Science (CPCI-S) --1990-present
  - Conference Proceedings Citation Index- Social Science & Humanities (CPCI-SSH) --1990-present
  - Book Citation Index– Science (BKCI-S) --2008-present
  - Book Citation Index– Social Sciences & Humanities (BKCI-SSH) --2008-present
  - Emerging Sources Citation Index (ESCI) --2015-present
  TOPIC: ((Accompan* OR Collaborat* OR Cooperat* OR Engag* OR Initiative* OR Integrat* OR Interact* OR Liaison OR Model* OR Partners OR Partnership* OR Team*) AND (Crime OR Crisis OR Crises OR Event OR Events OR Occur* OR Incident* OR Emergenc* OR Disturbance*) AND (Arrest* OR Custody OR “Law Enforcement” OR Police OR “Re-Arrest”) AND (“Mental Health Conditions” OR “Mental Health Crisis” OR “Mental Health Issues” OR “Mental Illness” OR “Mental Illnesses” OR “Psychiatric Crisis” OR “Psychiatric Emergencies” OR “Psychiatric Emergency” OR “Psychiatric Syndromes”))
  Timespan: All years. Indexes: SCI-EXPANDED, SSCI, A&HCI, CPCI-S, CPCI-SSH, BKCI-S, BKCI-SSH, ESCI.

## Additional file

Additional file 1: Contains the completed PRISMA-P for the protocol. (DOC 89 kb)

## Abbreviations

CCRs: Police Contact Control Rooms
CIT: Crisis Intervention Teams
CPS: Crown Prosecution Service
GRADE: Grading of Recommendations, Assessment, Development and Evaluations
L&D: Liaison and Diversion
PICO: Population/Participants, Intervention/Indicator, Comparator/Control, Outcomes
SMI: Severe mental illness
YOT: Youth Offending Team(s)

## Footnotes

1 Available at http://www.riskofbias.info/

2 Available via https://www.crd.york.ac.uk/PROSPERO/display_record.asp?ID=CRD42017057039
