# Supplementary material for: Effectiveness of current policing-related mental health interventions in England and Wales and Crisis Intervention Teams as a future potential model: a systematic review
Source: Syst Rev. 2017 Apr 17;6:85. doi: 10.1186/s13643-017-0478-7 (PMC5393040; doi:10.1186/s13643-017-0478-7)
Supplement: Additional file 1: — Contains the completed PRISMA-P for the protocol. (DOC 89 kb) [file 13643_2017_478_MOESM1_ESM.doc]

**PRISMA-P (Preferred Reporting Items for Systematic review and Meta-Analysis Protocols) 2015 checklist: recommended items to address in a systematic review protocol***

| Section and topic | Item No | Checklist item |
| --- | --- | --- |
| ADMINISTRATIVE INFORMATION | | |
| Title: |  |  |
| Identification | 1a | This report is the protocol for a systematic review of: Effectiveness of current Policing related Mental Health Interventions in England and Wales and Crisis Intervention Teams as a future potential model: A Systematic Review |
| Update | 1b | NA |
| Registration | 2 | The registration number for this review in PROSPERO International prospective register of systematic reviews is CRD42017057039 and it can be accessed via: https://www.crd.york.ac.uk/PROSPERO/display_record.asp?ID=CRD42017057039. |
| Authors: |  |  |
| Contact | 3a | Professor Eddie Kane (corresponding author)  Centre for Health and Justice  Institute of Mental Health  University of Nottingham  Triumph Road, NG7 2TU  Nottingham, UK  eddie.kane@nottingham.ac.uk  Emily Evans  School of Medicine  University of Nottingham  Nottingham, UK  emily.evans@nottingham.ac.uk  Farhad Shokraneh  Institute of Mental Health  University of Nottingham  Nottingham, UK  farhadshokraneh@gmail.com |
| Contributions | 3b | Kane and Evans developed the PICO, defined the resources and databases to be searched, and undertook the screening of results. Shokraneh developed the search strategies and will execute the searches. Kane is the guarantor of this work. |
| Amendments | 4 | NA |
| Support: |  |  |
| Sources | 5a | Review is being undertaken as part of work under the Police Knowledge Fund (HEFCE award number RR4809, recipient Eddie Kane) |
| Sponsor | 5b | HEFCE |
| Role of sponsor or funder | 5c | NA |
| INTRODUCTION | | |
| Rationale | 6 | In recent years there has been an increase in the level of investment related to intervention options for the police when encountering those with mental health problems. In England there are three commonly used but not necessarily commonly designed or operated interventions; Liaison and Diversion (L&D), Street Triage and specialist staff embedded in Police Contact Control Rooms (CCRs). In addition, the Crisis Intervention Team (CIT) model is currently being used elsewhere and there is some early interest in the approach amongst police forces. This review will address an evidence gap around these interventions providing a baseline of research evidence for those who commission and provide services for individuals experiencing mental ill health and who are in contact with the justice system. |
| Objectives | 7 | The objectives of the review are to investigate the effectiveness of current policing related mental health interventions in England and Wales. The review will identify and report research on the relevant policing interventions, nationally and internationally and consider, when possible, which interventions or aspects of those interventions that are effective, regarding criminal justice and health outcomes. |
| METHODS | | |
| Eligibility criteria | 8 | The key elements of the PICO are: persons with mental health problems, symptoms, or diagnoses who come into contact with the police; interventions involving partnership working between police and mental health nurses to divert those with mental health problems away from criminal justice processes; comparisons with control groups or areas where such interventions have not been introduced; outcomes concerning criminal justice and health outcomes.  The inclusion criteria requires any included studies to report an objective outcome measure(s) regarding offending or mental health, and to have an experimental or quasi-experimental design including a comparator group(s) or pre/post comparison. The review excludes PhD theses, papers in non-English languages and papers published prior to 1980. |
| Information sources | 9 | Twenty-nine relevant databases and sources have been selected which will be systematically searched for papers published after 1980. Keywords have been collected through experts’ opinion, literature review, controlled vocabulary, and reviewing the results of a primary scoping review to allow the above PICO to be developed. |
| Search strategy | 10 | G. PsycINFO via Ovid SP  1. (Accompan* or Collaborat* or Cooperat* or Engag* or Initiative* or Integrat* or Interact* or Liaison or Model* or Partners or Partnership* or Team*).ti,ab.  2. Crime/ OR Criminals/ OR Crises/ OR Emergency Management/ OR Crisis Intervention/ OR (Crime or Crisis or Crises or Event or Events or Occur* or Incident* or Emergenc* or Disturbance*).ti,ab.  3. Law Enforcement/ OR Police Personnel/ OR (Arrest* or Custody or "Law Enforcement" or Police or "Re-Arrest").ti,ab.  4. Exp Mental Disorders/ OR Mentally Ill Offenders/ OR Psychiatric Patients/ OR ("Mental Health Conditions" or "Mental Health Crisis" or "Mental Health Issues" or "Mental Illness" or "Mental Illnesses" or "Psychiatric Crisis" or "Psychiatric Emergencies" or "Psychiatric Emergency" or "Psychiatric Syndromes").ti,ab.  5. 1 AND 2 AND 3 AND 4  To be applied for the period 1980 onwards. |
| Study records: |  |  |
| Data management | 11a | Search results will be presented and managed in EndNote X7. This will be used to undertake the initial screen of the results, based on title and abstract. Papers which require a full text review will be gathered and managed electronically where possible. |
| Selection process | 11b | Studies returned in the search results will be initially screened on the title and abstract, with a second level screening undertaken on a sub-sample on the full text of the item. The reasons for exclusion will be noted for each record. This will be undertaken by two independent researchers (Kane and Evans). Disagreement will be resolved through discussion or recourse to a third party. |
| Data collection process | 11c | Of those studies included, following a full text review, the aims, methodological approach and findings will be gathered and reported in the review. This will be undertaken by Evans independently. |
| Data items | 12 | Research aims and questions, sample size and type, study design (including whether a comparison is used or a pre/post deign), and findings including whether findings or differences or statistically significant. Due to the limited and varied nature of the research evidence in this field, it is expected that these items will vary across the included studies. |
| Outcomes and prioritization | 13 | Objective outcome measure(s) regarding criminal justice or mental health including: improved assessment, referral and treatment (quality and timeliness) of those with mental illness; improved mental health outcomes (such as change in status or diagnosis as assessed by practitioners using valid measures) and service engagement; reduced use of Section 135/6 of the Mental Health Act; reduced demand on police forces and police officer time; increased demand on community mental health services; improved multi-agency working amongst those involved; reductions in reoffending. Any of these will be considered equally and are equally relevant. |
| Risk of bias in individual studies | 14 | In order to avoid publication bias and selective reporting the search conducted will include grey literature and unpublished reports. In addition, because research protocols in this area do not get formally registered, the authors will also aim to assess the results from different studies together and contact the authors for more information if required. Relevant studies will be assessed for risk of bias using the Cochrane tools for RCT and non-RCT designs, RoB 2.0 and ROBINS-I respectively (Available at: http://www.riskofbias.info/). These consider aspects of the study design and conduct. |
| Data synthesis | 15a | Where the study data allows it we will conduct data synthesis, using risk ratios for binary data, mean difference for continuous data, or effect estimates for summary effects data. It is expected however that due to the limited and varied nature of the research evidence in this field, it will not be possible to quantitatively synthesize findings across the included studies. The findings will be narratively synthesised. |
|  | 15b | NA |
|  | 15c | NA |
|  | 15d | Studies reporting the same outcomes measures, such as number of arrests, days in jail, day in treatment, will be compared and reported alongside each other. Studies reporting outcome measures on similar topics, such as recidivism or mental health treatment, will also be compared as far as possible. |
| Meta-bias(es) | 16 | As above, in order to avoid publication bias and selective reporting, the review will consider grey literature and unpublished reports and the authors will aim to assess the results from different studies together and contact the authors for more information if required. |
| Confidence in cumulative evidence | 17 | Where the nature of the studies permit it, A GRADE table of findings will be developed in order to support the identification of relevant results with regard to the aims of the study, research design, and outcome measures. As above the nature of the studies in the topic area may require such a table to be adapted. |

*** It is strongly recommended that this checklist be read in conjunction with the PRISMA-P Explanation and Elaboration (cite when available) for important clarification on the items. Amendments to a review protocol should be tracked and dated. The copyright for PRISMA-P (including checklist) is held by the PRISMA-P Group and is distributed under a Creative Commons Attribution Licence 4.0.**

*From: Shamseer L, Moher D, Clarke M, Ghersi D, Liberati A, Petticrew M, Shekelle P, Stewart L, PRISMA-P Group. Preferred reporting items for systematic review and meta-analysis protocols (PRISMA-P) 2015: elaboration and explanation. BMJ. 2015 Jan 2;349(jan02 1):g7647.*
